# Case report: Variable response to immunotherapy in ovarian cancer: Our experience within the current state of the art

**DOI:** 10.3389/fimmu.2022.1094017

**Published:** 2022-12-19

**Authors:** Nicoletta Provinciali, Marco Greppi, Silvia Pesce, Mariangela Rutigliani, Irene Maria Briata, Tania Buttiron Webber, Marianna Fava, Andrea DeCensi, Emanuela Marcenaro

**Affiliations:** ^1^ Division of Medical Oncology, Ente Ospedaliero (E.O.), Ospedali Galliera, Genoa, Italy; ^2^ Dipartimento di Medicina Sperimentale (DIMES), Università degli Studi di Genova, Genova, Italy; ^3^ Division of Pathology, Ente Ospedaliero (E.O.) Ospedali Galliera, Genoa, Italy; ^4^ Centre for Cancer Prevention, Wolfson Institute of Preventive Medicine, Barts and the London School of Medicine and Dentistry, Queen Mary University of London, London, United Kingdom; ^5^ IRCCS Ospedale Policlinico San Martino, Genova, Italy

**Keywords:** ovarian cancer, immunotherapy, avelumab, PD-1, PD-L1

## Abstract

Despite recent advances in ovarian cancer (OC) treatment, including the introduction of bevacizumab and PARP-inhibitors, OC remains a lethal disease. Other therapeutic options are being explored, such as immunotherapy (IT), which has been proved effective in many solid tumors. Findings about tumor-infiltrating cytotoxic and regulatory T cells, together with the expression of PD-1 on immune cells and of PD-L1 on tumor cells, gave the rationale for an attempt to the use of IT also in OC. We treated two patients with avelumab, an anti-PD-L1 monoclonal antibody, after the first line of chemotherapy: Patient A underwent 19 cycles of maintenance therapy with avelumab with a disease-free interval of 12 months, whereas patient B showed a slight progression of disease after only eight cycles. A higher PD-L1 expression in tumor cells of patient A was detected. She also underwent a genomic assessment that described the presence of a high Tumor Mutational Burden (TMB) and a status of Loss of Heterozygosity (LoH). This different response to the same treatment puts in evidence that some genomic and immune features might be investigated.

## Introduction

Ovarian cancer (OC) is the third most common but the first most lethal gynecologic malignancy all over the world, as it represents the fifth cause of death by cancer in women, with 21,750 new cases and 13,940 deaths estimated in the USA in 2020 (1).

The treatment of OC has always consisted in the combination of surgery and platinum-based chemotherapy (2). The therapeutic innovations in the past decade consisted in the introduction of bevacizumab (3) and, only recently, in the advent of PARP inhibitors (PARPis) (4).

However, despite the availability of these new therapeutical options, the prognosis for women affected by advanced OC is still poor. Therefore, other strategies, such as targeting specific molecules on cancer cells or harnessing the host’s immune system, need to be explored more carefully.

Immunotherapy (IT) based on the stimulation of the endogenous immune response against tumor cells is the last frontier in cancer treatments and it is now widely used in many solid tumors, with results so satisfactory that the natural history of some types of cancers, such as non-small cell lung carcinoma (NSCLC) and melanoma, has dramatically changed. The most common type of IT consists in the use of monoclonal antibodies, which can be directed against immunosuppressive receptors (ICI, immune checkpoint inhibitors), expressed not only by activated T cells but also by Natural Killer cells, such as PD-1 (5) (6), NKG2A (7), and CTLA4 (8) or against their ligands, such as PD-L1 (9), expressed by tumor and immune cells.

Based on this evidence, IT has now been included in the standard of care for several malignancies. The possibility of considering IT as a viable option also for OC is based on the finding that the presence of tumor-infiltrating lymphocytes is correlated with better survival, whereas the presence of regulatory T cells is a negative prognostic factor (10–12). In addition, the existence of several escape mechanisms exploited by OC cells to prevent T and NK cell–mediated attack strongly suggests a critical role for these adaptive and innate cells in OC immunosurveillance (13–17).

In addition, patients with the BRCA 1/2 mutation showed high expression of PD-1 on immune cells and of PD-L1 on tumor cells (18). Taken together, all this evidence has motivated the exploration of a possible application of IT also to OC. Several clinical trials exploring various immune-based strategies have been conducted for this purpose; single agent therapies including ICI, vaccines, monoclonal antibodies, adoptive cell therapy have shown modest effects (19, 20), but their combination in a synergistic treatment, directed toward either tumor cells or the immune microenvironment could lead to better clinical response, so further explorations are needed (21, 22).

Many phases II and III trials investigating IT in OC are still ongoing, mainly addressing recurrent disease and exploring various possible combination of IT with not only the standard of care, for example, ICI with chemotherapy and/or anti-angiogenic agents and/or PARPis, but also the association of different immune approaches (23).

Here, we report our experience with the use of IT in OC, reporting the cases of two patients who showed an almost opposite response to treatment, despite an almost overlapping therapeutic path. We will furthermore describe some histological, genetic, and molecular characteristics that diversify these patients in an attempt to identify potential predictive and prognostic factors of the response to IT.

## Case description

**Figure 1:**
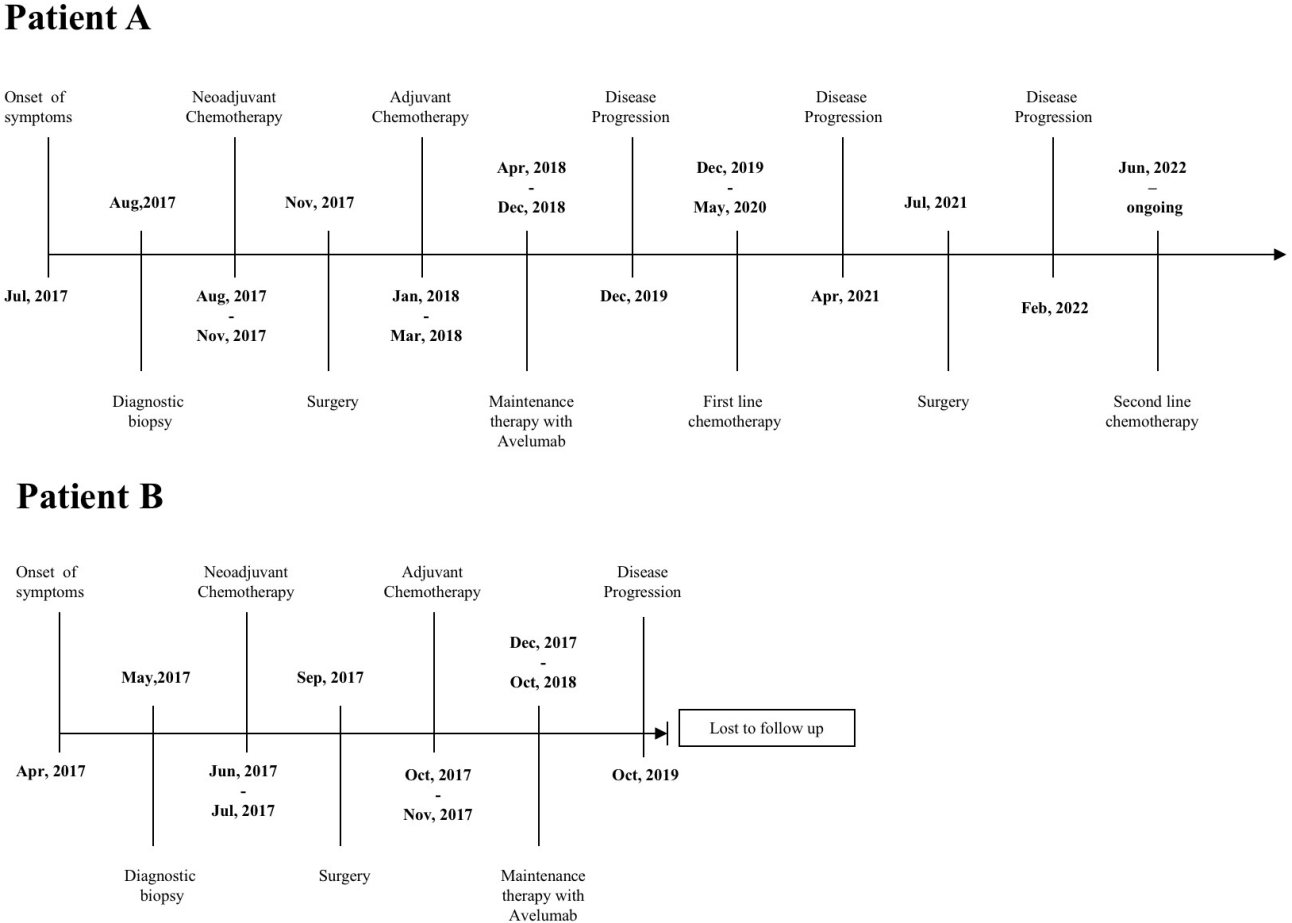
Visual snapshot of significant healthcare events of Patients A and B.

Patient A was a 71-year-old woman with a history of ocular glaucoma, whereas Patient B was a 75-year-old woman in good health; neither patient had oncological familiarity or gene mutations already known at the time of diagnosis. Details of the patient’s clinical course are outlined in **Figure 1**.

On 19 July 2017, Patient A presented with dyspnea and abdominal pain. A total body CT scan showed ascites, bilateral pleural effusion, and peritoneal nodules with inhomogeneous ovaries suggestive of cancer. Biopsy of peritoneum confirmed histological diagnosis of advanced high-grade serous OC (HGSOC).

Patient B was admitted to the Emergency Room for abdominal pain and constipation on 11 April 2017. A CT scan revealed the presence of suspicious ovaries, omental cakes, peritoneal nodules, and multiple pathological abdominal lymph nodes. Again, a peritoneal biopsy confirmed the diagnosis of malignancy of probable ovarian origin. For both the patients, the definitive diagnosis of HGSOC was subsequently confirmed by the histology performed following debulking surgery.

Both the patients were enrolled in JAVELIN Ovarian 100 trial, a phase III study comparing avelumab (anti–PD-L1 monoclonal antibody) in combination with chemotherapy followed by avelumab maintenance (arm A), or chemotherapy alone followed by avelumab (arm B), *versus* chemotherapy alone (arm C) in patients with previously untreated OC (24). Both the patients were randomized in the arm B of the trial.

Patient A underwent three cycles of neoadjuvant chemotherapy (carboplatin AUC 5 intravenously, every 3 weeks, with paclitaxel 80 mg/m^2^ weekly) from 24 August 2017 to 2 November 2017 with a partial response on both ovarian/peritoneal disease and pleural effusion. She was candidate for debulking surgery performed on 23 November 2017 and completed chemotherapy with three more adjuvant cycles from 18 January 2018 to 19 March 2018. The CT scan performed after the completion of chemotherapy showed a complete response. Thus, she started maintenance therapy with avelumab 10 mg/kg every 2 weeks for 19 cycles from 5 April 2018 to 11 December 2018. The treatment was then interrupted because of early termination of the trial due to ineffectiveness of therapy, as demonstrated by interim analysis. At that time, Patient A had still no evidence of disease; the first progression was recorded on December 2019 when a CT scan showed peritoneal lesions, with a disease-free interval of 12 months. She underwent second-line chemotherapy with carboplatin AUC 5 and Caelyx 30 mg/mq day 1 every 28 for six cycles from 9 December 2019 to 5 May 2020 and a CT scan performed after the treatment showed a complete response again. Disease free survival time was of 11 months, then a PET scan performed in April 2021 revealed the appearance of disease on the right adrenal gland which was surgically removed in July 2021. Patient was disease free until February 2022 when a PET scan showed a single peritoneal lesion. On 3 June 2022, Patient A started chemotherapy again with carboplatin AUC 5 day 1 plus gemcitabine 800 mg/m^2^ days 1 and 8 every 21, and she is undergoing treatment at the moment.

Patient B underwent three cycles of neoadjuvant chemotherapy (Carboplatin AUC 5 and paclitaxel 175 mg/m^2^ every 3 weeks) from 14 June 2017 to 26 July 2017, with partial response on peritoneum and mediastinal nodes and disease progression on ovary; surgery was performed on 4 September 2017, followed by three more cycles of adjuvant chemotherapy from 18 October 2017 to 29 November 2017 obtaining a complete response. Maintenance with avelumab was started on 29 December 2017. On April 2017, after eight cycles of therapy, CA 125 serum levels started to increase and a CT scan and a PET scan both confirmed the appearance of small peritoneal lesions. In consideration of a slight progression of disease against a subjective clinical benefit of the patient, it was decided to continue the therapy with avelumab, in accordance with the trial’s medical monitor and the patient, until 17 October 2018, for a total of 20 cycles, when the treatment was definitely stopped. In this case, the interruption of therapy was due to an evident disease progression in peritoneum, mediastinal, and abdominal lymph nodes. Shortly after, this patient was lost to follow up due to moving to Romania, her native country.

Faced with two such different responses despite a substantially overlapping and common therapeutic path, we tried to retrospectively analyze some histological and biological characteristics of the patients that could explain such a difference in treatment efficacy.

**Figure 2:**
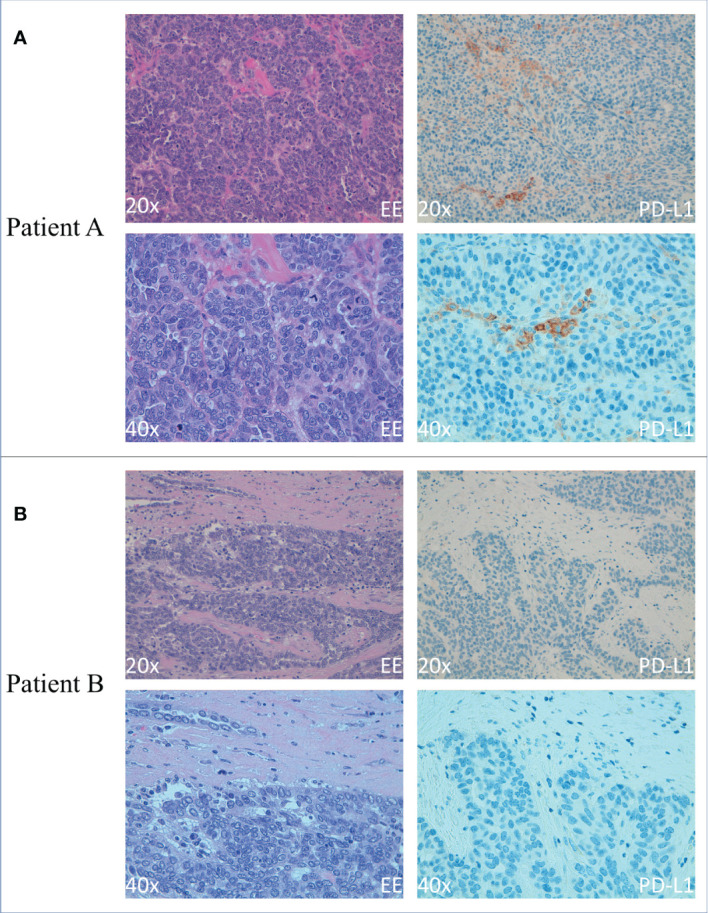
Immunohistochemical analysis of PD-L1 expression on OC cells from Patients A and B **(A)** Smaller (20×) (upper panels) and larger (40×) (lower panels) magnification of primary OC showing hematoxylin-eosin staining (left panels) and PD-L1+ tumoral cells (right panels) of Patient A **(B)** Smaller (20×) (upper panels) and larger (40×) (lower panels) magnification of primary OC showing hematoxylin-eosin staining (left panels) and PD-L1+ tumoral cells (right panels) of Patient B Scale bars in A and B are 100 μm.

Regarding the contribution of PD-1/PD-L1 blockade IT in the overall survival and progression-free survival of patients, it is of note that the most widely used biomarker with some prediction capabilities for the outcome of the treatment is PD-L1 expression in tumor biopsies. For this reason, we performed immunohistochemistry (IHC) analysis of this marker in tumor biopsies derived from Patients A and B before and after IT. Tumor proportion score (TPS) has been used to evaluate PD-L1 expression. In particular, PD-L1 expression was calculated as the percentage of tumor cells with membrane staining of any intensity for each core; the final score was calculated as the average of all available cores. Cases were considered positive for PD-L1 when ≥ 1% of the tumor cells expressed PD-L1. We observed a higher expression of PD-L1–positive tumor cells in Patient A. In particular, immunohistochemical staining was performed on 2-µm thick FFPE serial with an automated IHC staining system (Ventana BenchMark ULTRA, Ventana Medical Systems, Italy). Sequential IHC was performed on Ventana BenchMark ULTRA, using a ultraView Universal DAB detection Kit. Afterward, slides were incubated with VENTANA PD-L1 (SP263) CE IVD US EXPORT antibody. (**Figure 2**).

Evaluation of the peritumoral inflammatory infiltrate indicated an increase in infiltrating lymphocytes in Patient A compared with Patient B, mainly T cells (CD3^+^ cells) and a minor but detectable proportion of innate lymphocytes (NKp46^+^ CD3^−^ cells) (data not shown).

As it was not yet in use in clinical practice, neither patient had been tested for the BRCA 1/2 gene mutation. However, Patient A agreed to undergo FoundationOne^®^ CDx (**Table 1**), a validated Comprehensive Genomic Profile able to detect four classes of genomic alterations targeting the entire coding sequence of 324 cancer-related genes plus select introns from 36 genes frequently rearranged in cancer. The application of this test to patients with solid tumors may be particularly useful in identifying those that show some genetic alterations that could potentially make them susceptible to IT, such as the TMB, the Microsatellite Instability (MSI) or the LoH (25, 26).

**Table 1:** Genomic alterations of patient A by FoundationOne^®^ CDx test.

Genomic Signatures	Loss of Heterozygosity score - 20.5 %Tumor Mutational Burden - 10 Muts/MbMicrosatellite status -MS-Stable
**Gene Alterations**	TP53 V157del PRKCI amplification TERC amplification

The test showed the presence of an increased TMB, which is now a recognized factor in the identification of tumors, which are most responsive to both anti–PD-1 and anti–PD-L1 agents (27). On the contrary, the tumor was also characterized by Microsatellite Stability (MSS), which is a well-known negative predictive factor of response to ICIs, differently from MSI. This evidence has been validated above all in colorectal cancer for which the determination of the state of MSS is carried out routinely in common clinical practice (28).

## Discussion

Regarding these two patients treated with the anti–PD-L1 monoclonal antibody avelumab, after the first line of chemotherapy, Patient A showed progression after 12 months and 19 cycles, whereas Patient B showed a slight progression of disease after only eight cycles. Interestingly, our data showed that Patient A tumor, which showed a better response to IT, was characterized by a high expression of PD-L1. In contrast, Patient B, who showed a worse outcome and a weak advantage from IT, was consistently negative for PD-L1. While PD-L1 was proven to be remarkable as a target for IT in melanoma (29) and lung cancer (30), its importance in OC is yet to be proven. These data confirm the importance of analysis of PD-L1 expression for the selection of therapeutic approach.

Nevertheless, PD-L1, as a biomarker for clinical diagnostic, shows some limitations, including differences among PD-L1 assays and scoring methods, as each method of PD-L1 detection has been developed by a different pharmaceutical company and the protocols and thresholds for positivity are associated with the methodology used in each trial (31–33). Other concerns to be considered are the dynamic and heterogeneous PD-L1 expression within tumors, which might differ between the biopsy and the rest of the tumor tissue, the time gap between the biopsy and therapeutic decisions (34–38).

In addition, in the case of patients treated with IT as a second or further line of treatment, there is a time gap between the diagnosis and the clinical decisions during which intermediate treatments such as conventional chemotherapy may alter PD-L1 expression in tumors. In fact, as in the case of the two patients discussed here, if the disease is too bulky to undergo upfront surgery, a first biopsy is obtained for diagnostic purposes but the definitive histological examination on the surgical tissue takes place after the administration of neo-adjuvant chemotherapy. Furthermore, it is possible, in case of disease recurrence, that new histological tissue is obtained from a metastatic lesion after further chemotherapy or other maintenance therapy such as bevacizumab or PARPis. The dynamic regulation of PD-L1 expression could explain clinical cases showing that patients diagnosed as tumor PD-L1 negative show objective responses to an anti–PD-L1 antibody as a second-line treatment (39).

Another concern is related to the heterogeneous nature of the tumor, which may affect PD-L1 quantification depending on the origin of the biopsy (primary tumor or metastasis), the degree of intratumoral heterogeneity and the sampling methodology (biopsy or tumor resection) (40). In conclusion, these data show that inconsistent response to IT in OC might be related to significant differences in PD-L1 expression in the tumor tissue that may humper its predictive potential. However, in OC, neither PD-L1 expression is always considered before the start of the therapy nor a consistent standardization for PD-L1 testing has been defined to obtain clear and comparable results.

Furthermore, considering that the immune infiltrate within tumors has proved to be very powerful in the prognostic stratification of patients, much attention should also be paid to its predictive value (41).

With a view to a possible future introduction of IT for the treatment of OC, considering the high cost of these therapies and the risk of immune-related adverse events during therapy, it will be necessary to identify the best combination of biomarkers that would facilitate the identification of potential responders and non-responders before the start of IT and a standardized evaluation of PD-L1 expression should become part of the routinely evaluated biomarkers in OC to better identify possible responders.

## Data availability statement

The original contributions presented in the study are included in the article/supplementary material. Further inquiries can be directed to the corresponding authors.

## Ethics statement

The studies involving human participants were reviewed and approved by ethics committee of the Liguria Region, Genova, Italy (n. 326/2018 and n127/2022-DB id12223 and B9991010 trial - JAVELIN Ovarian 100(EudraCT: 2015-003239-36). The B9991010 trial was supported by Pfizer, as part of an alliance between Pfizer and Merck (CrossRef Funder ID: 10.13039/100009945). The patients/participants provided their written informed consent to participate in this study. Written informed consent was obtained from the individual(s) for the publication of any potentially identifiable images or data included in this article.

## Author contributions

NP, MG, and SP interpreted data, and wrote the article; MR performed immunohistochemical analyses; NP, MR, and AD provided samples and managed patient’s profile; TW managed patient’s profile; IB and MF revised the article; EM financed, designed, interpreted data, and wrote the article. All authors contributed to the article and approved the submitted version.
